# PUB1/359: The Use of the Internet for CME Purposes in Turkey

**DOI:** 10.2196/jmir.1.suppl1.e104

**Published:** 1999-09-19

**Authors:** H Yaman, A Kut

**Affiliations:** ^1^Ankara Numune Teaching Hospital, AnkaraTurkey

**Keywords:** Internet, CME, Primary Care, Electronic Publishing

## Abstract

While continuing medical education (CME) is receiving increasing attention from medical educators and health administrators world-wide, many efforts need to be made to improve its performance and overall effectiveness. CME has depended primarily on periodic courses and conferences. High costs and distant location make CME journals an alternative to these events. The Turkish Medical Association is publishing a journal for CME purposes called STED. By this way, every month, 9000 exemplars of each edition are distributed mainly to primary care physicians in the whole of Turkey. To make the journal also accessible to non-subscribers and professionals, who live outside Turkey, English abstracts of articles published in STED are going to be prepared and published soon by Internet services (www.ato.org.tr). By increasing the use of computer-assisted teaching and modern telecommunications, in the near future, the costs of CME can be reduced and its effectiveness improved.

